# Cortisol in fish scales remains stable during extended periods of storage

**DOI:** 10.1093/conphys/coae065

**Published:** 2024-09-19

**Authors:** Christina O’Toole, Philip White, Conor T Graham, Caitlin Conroy, Deirdre Brophy

**Affiliations:** Marine and Freshwater Research Centre, ATU Galway City, Atlantic Technological University (ATU), Dublin Road, Galway H91 T8NW, Ireland; Fisheries Ecosystem Advisory Services, Marine Institute, Rinville, Oranmore, Co. Galway H91 R673, Ireland; Marine and Freshwater Research Centre, ATU Galway City, Atlantic Technological University (ATU), Dublin Road, Galway H91 T8NW, Ireland; Marine and Freshwater Research Centre, ATU Galway City, Atlantic Technological University (ATU), Dublin Road, Galway H91 T8NW, Ireland; Marine and Freshwater Research Centre, ATU Galway City, Atlantic Technological University (ATU), Dublin Road, Galway H91 T8NW, Ireland; Marine and Freshwater Research Centre, ATU Galway City, Atlantic Technological University (ATU), Dublin Road, Galway H91 T8NW, Ireland

**Keywords:** Atlantic salmon (*salmo salar*), bioindicator, fish scale archive, stress response

## Abstract

Measurement of cortisol in fish scales is attracting considerable attention as a non-invasive indicator of chronic stress in wild populations. For many fish species of management and conservation interest, extensive scale collections exist that could provide extended records of individual stress responses, by combining cortisol measurements with life history information. However, it is not yet known how well cortisol is preserved in the scale during storage. To investigate the stability of scale cortisol, we accelerated potential degradation by storing scales from an individual farmed Atlantic salmon (*Salmo salar*) in an oven at 50°C for between 2 and 12 weeks. We found no significant relationship between scale cortisol concentration and either storage time or storage temperature. Cortisol concentrations in scales from the same fish were consistent (18.54–21.82 ng. g^−1^; coefficient of variation (CV) = 3.6%), indicating that scale cortisol can be reliably quantified, even in scales stored for varying periods of time or under different conditions. We also examined the effects of storage in real time using Atlantic salmon scales that were stored in paper envelopes at room temperature for between 3 and 32 years and found no significant relationship between scale cortisol concentration and storage time. Scale cortisol concentrations ranged from 4.05 to 135.37 ng.g^−1^ and levels of between-individual variability were high (CV = 61%). Given that scale cortisol does not degrade during long-term storage, historical scale collections and associated data describing fish life histories could potentially be used to develop bioindicators of physiological responses in fish populations. Further research is needed to understand scale cortisol variability and its biological relevance.

## Introduction

In the field of conservation physiology, indicators of how organisms respond to stressors in their environment are increasingly important for providing mechanistic understanding of anthropogenic impacts and identifying critical thresholds for ecosystem restoration and management (Cooke *et al.,* 2013, 2023). The most widely used indicators of physiological stress in vertebrates include measurements of glucocorticoid hormones, typically cortisol and cortisone, in various matrices (Newediuk and Bath, 2023). In fish, cortisol in scales provides a particularly attractive biomarker because unlike cortisol in plasma, concentrations are not affected by stress experienced during sampling (Sadoul and Geffroy, 2019). Cortisol accumulates over time in the scale, providing a better indicator of chronic stress than cortisol in plasma or other matrices with a more rapid turnover (Aerts *et al.,* 2015; Laberge *et al.,* 2019; Kennedy and Janz, 2023a). Several recent studies have demonstrated that cortisol is detectable in scales across a range of species (Carbajal *et al.,* 2019b; Roque D’Orbcastel *et al.,* 2021; Vercauteren *et al.,* 2022; O’Toole *et al.,* 2023) and that concentrations remain elevated after exposure to stress (Hanke *et al.,* 2019; Laberge *et al.,* 2019; Carbajal *et al.,* 2019a; Goikoetxea *et al.,* 2021; Weirup *et al.,* 2021). In addition, it is possible to quantify scale cortisol non-lethally (Kennedy and Janz, 2023b). For these reasons, scale cortisol is attracting considerable attention as a potential tool for monitoring responses of wild populations to environmental change and habitat pressures.

Scales can be sampled with relative ease and stored dry without the need for preservatives or specialist equipment. They contain information about individual age and growth rates in their microstructure and so are routinely collected as part of monitoring programmes for many fish populations (Panfili *et al.,* 2002; King, 2007). Many fisheries organizations around the world hold extensive multi-decadal collections of scales for species of management and conservation interest (Desjardins *et al.,* 2006; Izzo *et al.,* 2016; Tray *et al.,* 2020) whilst museum collections hold scale samples from pre-industrial and even pre-historical time periods (Disspain *et al.,* 2016; Guiry and Hunt, 2020). Contemporary collections in particular, are often accompanied by data describing the life history of the fish (e.g. age, sex, reproductive status, environmental conditions at sampling location) that may contribute to variability in cortisol production (Mommsen *et al.,* 1999). These collections could therefore be used to examine spatial and temporal factors that contribute to variation in scale cortisol and to establish species- and area-specific baselines; an important pre-requisite to applying scale cortisol as a bioindicator (Carbajal *et al.,* 2019b). However, it is not yet known if cortisol remains stable in the scale during extended periods of storage. It is important to address this uncertainty in order to unlock the potential value of fish scale collections as extended records of individual responses to environmental change.

This study aimed to establish if cortisol in the scales of Atlantic salmon (*Salmo salar* L.) remains stable during storage or degrades over time. According to the Arrhenius equation, the rate of any chemical reaction is temperature-dependent, approximately doubling for each 10°C increase in temperature (Arrhenius, 1889). Accelerated stability studies take advantage of this property by measuring degradation at elevated temperatures to estimate how much degradation would be likely to take place during a longer period of storage at a lower temperature (Kirkwood, 1977; Waterman and Adami, 2005). We carried out an accelerated stability test by holding scales from an individual farmed salmon in an oven at 50°C for between 2 and 12 weeks. In addition, we examined the effects of storage in real time using scales that were stored in a scale collection for between 3 and 32 years. We examined the relationships between storage time and scale cortisol concentration for evidence of cortisol degradation. The results provide an important baseline for future applications of scale cortisol analysis to historical scale collections.

## Materials and Methods

### Collection of contemporary scales

One farmed Atlantic salmon was obtained fresh and whole from a fish shop. Cortisol concentrations can vary in scales from different body locations (Laberge *et al.,* 2019), so the sampling location is an important consideration for scale cortisol analysis. For this analysis, scales were carefully scraped from both sides of the fish, in the region behind the dorsal fin and above the lateral line. Standardized protocols used in routine monitoring of Atlantic salmon populations recommend that scales are sampled from this location on fish as they are less likely to be regenerated and will provide the most reliable measurements of growth (ICES, 1982; Shearer, 1992). Additionally and importantly, scale collections held by fisheries institutions, including the one accessed for this study, predominantly contain scales sampled from this body region. Sixteen samples of scales were placed into individual acid-free paper envelopes, the type that is routinely used to store archived scales (Tray *et al.,* 2020).

### Collection of archived scales

Atlantic salmon scales were taken from the scale collection held at the Marine Institute’s Newport Research Facility in County Mayo, Ireland (Ó’Maoiléidigh *et al.,* 2020). These scales were from Atlantic salmon that were reared in a hatchery and released into the Burrishoole river system as smolts. They were caught in traps on the river and sampled for scales on their return migration after one winter at sea. All scales were taken from the standard sampling location, behind the dorsal fin on the left flank, 2–3 rows above the lateral line (Shearer, 1992) and stored at room temperature in acid-free paper envelopes (Tray *et al.,* 2020). For this study, scales of 20 fish from each of 6 years (1989, 1998, 1999, 2001, 2010, 2018) were taken from the collection (*n* = 120).

### Accelerated stability test using contemporary scales

To simulate an extended storage period, we held scales at 50°C for a period of up to 12 weeks, which, according to the Arrhenius equation, is approximately equivalent to 96 weeks at 20°C (room temperature) or 1536 weeks at −20°C (frozen storage) (assuming a doubling of the rate of reaction for each 10°C increase in temperature; Waterman and Adami, 2005). At the beginning of the experiment (week 0), two scale samples were placed in paper envelopes and held at room temperature. Two scale samples were weighed and placed in crucibles into a chamber furnace (Carbolite Gero ELF 11/14B) at 50°C. The remaining scale samples were placed in the freezer. Every two weeks (Weeks 2, 4, 6, 8 and 10), two scale samples were removed from the freezer, brought to room temperature, weighed and transferred to the furnace in a crucible. Two scale samples remained in the freezer for the 12-week duration. At Week 12 all scale samples were prepared for cortisol analysis as described below.

### Scale preparation for cortisol analysis

Using clean, dry equipment, archived scales were carefully removed from their envelopes and placed in ultrapure water (18.2 mΩ) for a minimum of 5 min. On a dissection microscope, scales were gently scraped with a scalpel whilst immersed in ultrapure water to remove mucous and residue and allowed to air dry. Scales were examined to ensure that no newly constituted replacement scales were used. Several scales from each envelope were cut with a scalpel to excise the portion of scale that was formed during the marine phase (demarcated by the freshwater exit check in the scale microstructure). This was to facilitate a comparison of scale cortisol and growth during the marine phase as part of a separate study. Excised scale segments were carefully cut into small pieces using a scalpel, placed in pre-weighed centrifuge tubes and the scale sample was weighed. The target weight of each sample for analysis was 10 mg. Actual sample weights ranged from 7.9–16.1 mg with an average weight of 10.6 mg (±1.3 SD). A previous study confirmed that scale cortisol can be reliably measured with these low weights of scale material (O’Toole *et al.,* 2023). Contemporary scales were processed in the same way but the entire scale was used without excising the marine portion.

### Cortisol analysis

Extraction and analysis of scale cortisol followed the procedure described in O’Toole *et al.* (2023) and adapted from Aerts *et al.* (2015). A reference material was fabricated by cleaning and cutting the scales (8.63 g) from a farmed adult salmon and adding a solution containing 76 ng.g^−1^ of cortisol (Cerilliant) and stored at −18°C prior to analysis. Each extraction batch included 14 scale samples, a procedural blank and 10 mg of the reference scales.

One hundred milligrammes of an internal standard containing 155.4–157.3 ng.g^−1^ cortisol-d4 (Cerilliant) was added dropwise by weight to each sample and standard followed by 8 ml of ROMIL Methanol 215 SpS (>99.9% assay). Samples were vortexed for 30 s, placed on a shaker at 800 osc/min for 1 h at ambient temperature and centrifuged for 10 min at 3260 *g*. The supernatant was pipetted into glass universal tubes and evaporated to almost dryness under nitrogen at 60°C until ~0.5 ml remained. Samples were reconstituted in 5 ml of H_2_O/MeOH (80:20, v/v). GracePure SPE C18-Max (500 mg, 6 ml) solid phase extraction (SPE) columns were conditioned on vacuum with 3 ml MeOH followed by 3 ml of ultrapure water. Samples were loaded using glass Pasteur pipettes, followed by 4.5 ml of H_2_O/MeOH (65:35, v/v). The samples were eluted with 3.5 ml of H_2_O/MeOH (20:80, v/v) and evaporated to almost dryness (~0.5 ml) at 60°C under nitrogen before being transferred to gas chromatography (GC) vials. Any sample with a volume less than the quarter delineation on the GC vial was topped up with H_2_O/MeOH (20:80, v/v). Equipment was cleaned and acid-washed between samples to prevent cross-contamination.

**Table 1:** Summary results from the GCMS analysis of cortisol concentrations in scales from an individual farmed adult Atlantic salmon (contemporary scales) that were exposed to different storage conditions for a period of 12 weeks and from 120 scales from wild-caught adult Atlantic salmon that were stored dry in paper envelopes at room temperature for 3–32 years (archived scales). The contemporary scales were stored in a freezer (−20°C), at room temperature (20°C) or in the oven (50°C) for the period indicated. Confidence interval is abbreviated to CI. Values below the LOD of 3.88 ng.g^−1^ cortisol are excluded from the calculations

**Group**	***n***	***n* above LOD**	**Mean cortisol ng.g** ^**−1**^ **(± 95% CI)**	**Min-max cortisol ng.g** ^**−1**^
*Contemporary scales*				
Frozen 12 weeks	2	2	20.01 (±0.39)	19.63–20.40
Frozen 10 weeks, oven 2 weeks	2	2	20.33 (±0.60)	19.73–20.93
Frozen 8 weeks, oven 4 weeks	2	2	20.57 (±0.12)	20.45–20.69
Frozen 6 weeks, oven 6 weeks	2	2	19.61 (±0.56)	19.05–20.17
Frozen 4 weeks, oven 8 weeks	2	2	19.00 (±0.45)	18.54–19.45
Frozen 2 weeks, oven 10 weeks	2	2	20.15 (±0.05)	20.09–20.20
Oven 12 weeks	2	2	19.73 (±0.43)	19.30–20.16
Room temperature 12 weeks	2	2	20.82 (±1.00)	19.82–21.82
*Archived scales*				
Stored 32 years (1989–2021)	20	15	56.83 (±6.95)	12.09–104.63
Stored 23 years (1998–2021)	20	19	41.57 (±7.61)	5.96–119.21
Stored 22 years (1999–2021)	20	19	49.46 (±6.33)	4.88–119.01
Stored 20 years (2001–21)	20	16	63.36 (±7.76)	4.05–109.23
Stored 11 years (2010–21)	20	20	50.21 (±7.53)	9.09–135.37
Stored 3 years (2018–21)	20	18	49.80 (±8.07)	6.08–134.85

Samples and standards were analysed on an Agilent 6890 N Network GC system with an Agilent Tech 5975 Mass Selective Detector with helium as the carrier gas and a Trajan (SGE) capillary GC column HT8 (25 m × 0.2 mm, 0.25 μm film thickness). The 189.1 m/z ion was used to identify cortisol, and the 306.1 m/z ion was used to identify cortisol-d4. The gas chromatography mass spectrometry (GCMS) was calibrated using a series of standard solutions containing cortisol (2–2000 ng.g^−1^) and 100 mg of the internal standard. The ratio of the cortisol and cortisol-d4 peak areas recorded by the instrument was plotted against the ratio of cortisol concentration to internal standard in the calibration standards. During analysis of the samples, the calibration plot was used to convert recorded cortisol:cortisol-d4 peak area ratios to cortisol concentration (ng.g^−1^) based on linear regression. The reference scales and system suitability standards were used to check instrument consistency across runs. The limit of detection (LOD) of the GCMS was calculated from the procedural blank concentrations. Cortisol values that were below the LOD of 3.8 ng.g^−1^ were excluded from the analysis. This resulted in the removal of data for 13 of the archive scale samples.

### Statistical analysis

For the contemporary scales, ordinary least squares (OLS) linear regression was used to model the relationship between scale cortisol concentration and number of weeks held in the oven at 50°C. The scales that were held in the freezer for 12 weeks were used as the baseline (0 weeks at 50°C). To model the relationship between cortisol concentration and storage temperature, a second OLS linear regression was fit to data from the six scale samples that had been stored under constant conditions for the 12-week period (frozen, at room temperature or in the oven).

For the archived scales, OLS linear regression was used to model the relationship between scale cortisol concentration and years spent in storage. Cortisol concentrations were square root transformed to achieve a normal distribution. In each case, a regression slope that was significantly different to zero would indicate that cortisol concentrations were altered during storage. A general linear model was used to examine the combined effects of years spent in storage and sex on cortisol concentrations. The best fitting model was selected based on Akaike Information Criteria (AIC) values and loglikelihood tests.

Percentage CVs of the cortisol concentrations were calculated according to the equation: $\frac{\sigma }{\mu}\ast 100$ where $\sigma$ is the standard deviation and $\mu$ is the mean. The CV of cortisol concentrations in the contemporary scales provided a measure of within-individual variation that could arise due to differences in cortisol concentrations between scales, methodological variability and changes introduced during storage. The CV of the archived scale measurements reflected between-individual variability in scale cortisol concentrations in addition to methodological variability and changes introduced during storage.

The statistical analysis was carried out using R version 4.3.1 in R Studio version 2023.06.1 + 524. The ggplot2 package (Wickham, 2016) was used for data visualization. An alpha level of 0.05 was used for tests of significance.

## Results

Cortisol concentrations ranged from 18.54 to 21.82 ng.g^−1^ in the contemporary scales and from 4.05 to 135.37 ng.g^−1^ in the archived scales, after removal of values below the LOD (Table 1). The percent CVs were 3.9% and 61% for the contemporary and archived scales, respectively, showing that repeated measurements from the same scale were consistent and within-individual variability was far exceeded by between-individual variability.

**Figure 1:**
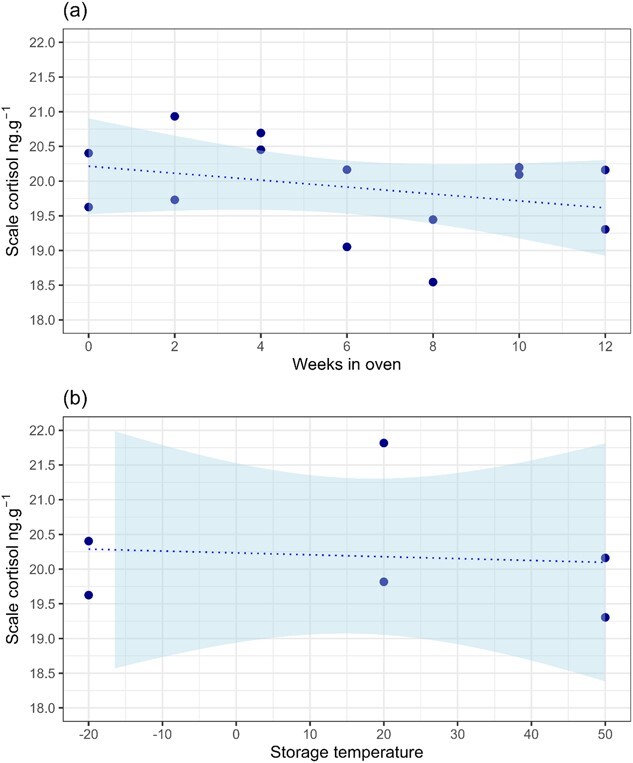
Cortisol concentration (ng.g^−1^) in scales from an individual farmed salmon held in an oven at 50°C for between 0 and 12 weeks. (a) shows the relationship between scale cortisol and number of weeks in the oven (R^2^ = 0.1, *P* = 0.28). (b) shows the relationship between scale cortisol and storage temperature for scales that were held in the freezer, at room temperature or in the oven for the full 12-week period (R^2^ = 0.009, *P* = 0.86). The dashed lines are the linear regression fits, shading indicates the 95% confidence limits of the model predictions.

**Figure 2:**
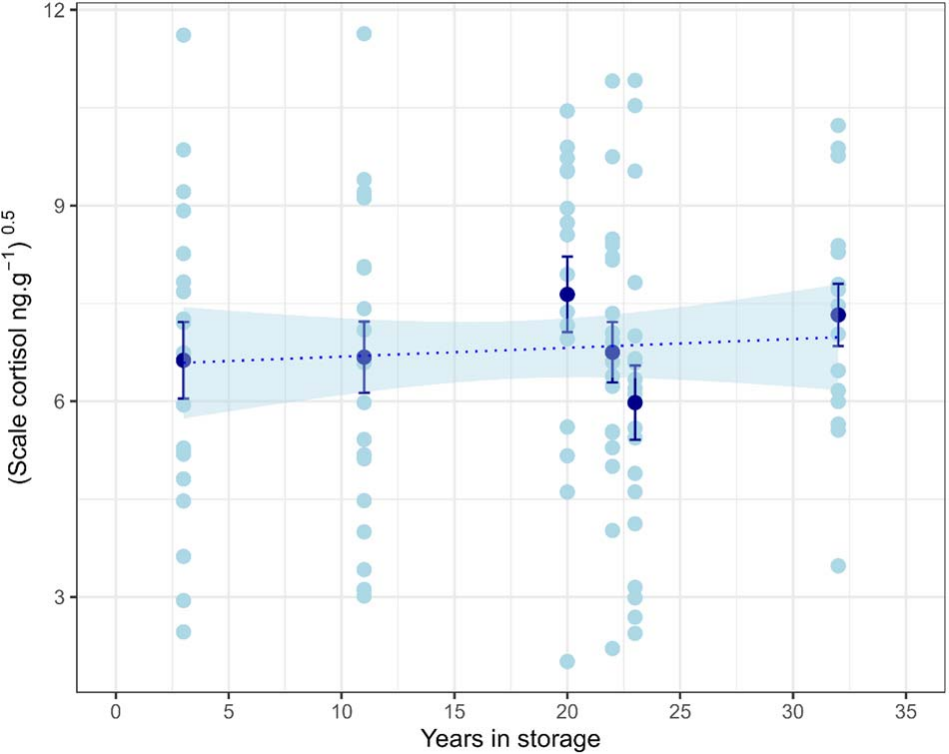
Cortisol concentration (ng.g^−1^)^0.5^ in scales of 107 wild-caught adult salmon sampled between 1989 and 2018 and analysed in 2021. Lightly shaded points indicate the individual measurements, dark shaded points indicate the mean values for each year. Whiskers indicate the 95% confidence intervals of the mean values. The dashed line is a linear regression of the relationship between cortisol concentration and years in storage (R^2^ = 0.003, *P* = 0.58); shading indicates the 95% confidence limits of the model predictions. The square root transform was applied to normalize the distribution of the data for regression analysis. Thirteen cortisol measurements that were below the limit of detection of 3.88 ng.g^−1^ were excluded from the plot.

The regression analyses confirmed that cortisol concentration in the contemporary scales was not affected by the length of time held at 50°C (Fig. 1a; R^2^ = 0.1, *P* = 0.28) or by temperature conditions during 12 weeks of continuous storage (Fig. 1b; R^2^0.009, *P* = 0.86). For the archived scales, there was no significant linear relationship between cortisol concentration and the number of years held in storage (Fig. 2; R^2^ = 0.003, *P* = 0.58). The relationship remained insignificant if machine values below LOD were included or imputed using robust linear regression following the method of Helsel (1990) and Higgins *et al.* (2013). When these values were included using either approach, the response variable was not normally distributed and so the regression based on data above LOD was preferred.

Inclusion of sex, years spent in storage or their interaction in a general linear model of scale cortisol concentration did not improve the fit relative to the null model. We concluded that scale cortisol concentration did not show any significant variation between the sexes or between years.

## Discussion

Our results show that cortisol levels in Atlantic salmon scales remain stable during extended periods of storage. The accelerated stability test used an elevated temperature of 50°C to mimic storage of up to 96 weeks at room temperature and found no significant relationship between scale cortisol concentration and either storage time or storage temperature. Similarly, for scales held in an archive for between 3 and 32 years, there was no relationship between scale cortisol concentration and storage time and no evidence that cortisol degraded during long-term storage. Cortisol measurements had high repeatability (CV = 3.6%) across scales from the single fish used in the stability test, indicating that scale cortisol can be reliably quantified, even in scales stored for varying periods of time or under different conditions. In light of these findings, the value of fish scale collections for generating extended time-series of physiological indices warrants further investigation.

Although temporally stable, cortisol concentrations in the archived scales were highly variable (CV = 61%) and were at the higher end of the range reported in other species (<3.8–135.37 ng.g^−1^). In general, scale cortisol concentrations vary by orders of magnitude between and within species. Vercauteren *et al.* (2022) recorded mean concentrations as low as 0.0034 ± 0.0046 ng.g^−1^ in scales from wild-caught dab (*Limanda limanda*), whilst at the other extreme, Goikoetxea *et al.* (2021) reported mean concentrations of 2100 ± 300 and 21 000 ± 6300 ng.g^−1^ in scales from juvenile European sea bass (*Dicentrarchus labrax*) reared at 16°C and 21°C, respectively. Hanke *et al.* (2020) observed that scale cortisol levels (0.04–0.21 ng.g^−1^) in milkfish (*Chanos chanos*) from commercial mariculture systems (held at ~31°C) were up to 100 times higher than those recorded by Hanke *et al.* (2019) in the same species reared under laboratory conditions (0.0014 ± 0.001 ng.g^−1^ at 26°C; 0.0020 ± 0.001 ng.g^−1^ at 33°C). In contrast, Weirup *et al.* (2021) reported scale cortisol concentrations for adult rainbow trout from commercial aquaculture farms (0.008–0.035 ng.g^−1^) that were more than an order of magnitude lower than control levels recorded in a laboratory study by Kennedy and Janz (2023a) (~0.49–2.2 ng.g^−1^) and 100 times lower than those reported by Kennedy and Janz (2022) (mean of control group ~20 ng.g^−1^). Species-specific differences, temporal and ontogenetic variation in energetic demands as well as methodological differences between studies may all contribute to the widely varying reported levels of cortisol in fish scales (Carbajal *et al.,* 2019b; Vercauteren *et al.,* 2022). The wild-caught adult Atlantic salmon used in this study were sampled at the end of a long-range spawning migration from offshore feeding grounds to their natal rivers. This period is intensely demanding physiologically, and plasma cortisol levels are known to rise during acclimation to fresh water (McCormick, 2001; Flores *et al.,* 2011). This could explain the relatively high concentrations of cortisol in the scales of these fish. Before scale cortisol can be widely adopted as an indicator of physiological stress, better knowledge is needed of the mechanisms driving variability in scale cortisol and the biological relevance of that variability.

Since Aerts *et al.* (2015) first proposed scale cortisol as an indicator of chronic stress in fish, various modifications of their original protocol have appeared in the literature. Scale cleaning prior to analysis has been conducted using water (Aerts *et al.,* 2015; Laberge *et al.,* 2019; Lebigre *et al.,* 2022), isopropanol (Carbajal *et al.,* 2019a, 2019b; Britton *et al.,* 2023) and methanol (Kennedy and Janz, 2022, 2023a, 2023b). Carbajal *et al.* (2018) observed that scale cortisol concentrations decreased after washing with water but not with isopropanol and suggested that water could leach endogenous cortisol from the scale as has been reported for hair (Hamel *et al.,* 2011), although they also acknowledged that the difference may have occurred because water was more effective than isopropanol at removing cortisol contamination from skin mucus. Methods used to break up the scale prior to cortisol extraction include cutting with scissors (Aerts *et al.,* 2015; Samaras and Pavlidis, 2022; O’Toole *et al.,* 2023), mechanical homogenization using a ball mill (Carbajal *et al.,* 2019a; Weirup *et al.,* 2021; Kennedy and Janz, 2023a, 2023b) and freeze drying (Roque D’Orbcastel *et al.,* 2021), although Laberge *et al.* (2019) found no differences in cortisol concentrations between scales that were homogenized prior to extraction and those that were left intact. The techniques used to analyse scale cortisol include ultra-performance liquid chromatography tandem mass spectrometry (Aerts *et al.,* 2015; Weirup *et al.,* 2021; Lebigre *et al.,* 2022), gas chromatography (this study, O’Toole *et al.,* 2023) radioimmunoassay (Laberge *et al.,* 2019) and enzyme immunoassay (Kennedy and Janz, 2023a, 2023b). Within a single study, repeated analysis of reference scales can confirm the repeatability of cortisol measurements and ensure that no substantial variation is introduced during sample preparation. However, greater standardization and cross-calibration of methodologies are needed to enable comparison between studies.

To the best of our knowledge, we are the first to investigate the effects of storage on the concentration of cortisol in fish scales. However, several previous studies have examined how storage impacts cortisol in mammalian hair. Cortisol is detectable in archaeological hair samples (Webb *et al.,* 2010) suggesting that it is stable over prolonged time periods and some authors report that storage of hair at room temperature for up to 1 year has no effect on cortisol levels (Macbeth *et al.,* 2010; González-de-la-Vara *et al.,* 2011). However, other lines of evidence show that cortisol in hair does degrade after multiple years (Azevedo *et al.,* 2019) or decades (Stewart *et al.,* 2018) in storage. Marked declines are observed after exposure to UV light (Wester *et al.,* 2016) or chemical washing (Hoffman *et al.,* 2014). Although hair is composed of keratin rather than collagen, both matrices are thought to accumulate cortisol through similar mechanisms: by diffusion from the blood (Russell *et al.,* 2012) and local production in hair follicles (Ito *et al.,* 2005) or the cells associated with fish scales (Samaras and Pavlidis, 2022). It has been suggested that the mineralization process could influence how scales incorporate steroid hormones (Kennedy and Janz, 2023a), which might also produce differences in stability during storage. Better knowledge of how cortisol is bound within the scale is needed to evaluate the potential for it to be altered during the life of the fish and post-mortem. In any case, the way scales are typically stored (in paper envelope, at room temperature and away from direct sunlight) appears to be appropriate for preserving cortisol over decadal time scales.

The stability of cortisol in scales raises exciting possibilities for using historical scale collections to develop bioindicators of physiological responses in wild populations over short- and long-term timescales. The extent to which scale cortisol measurements can support conservation and management decisions will depend on the availability of associated life history information, given that changes in cortisol may reflect metabolic processes that are unrelated to stress. Scale collections that support fisheries monitoring programmes are often accompanied by substantial datasets describing individual phenology, growth, maturation and genetics (Jasper *et al.,* 2013; Tréhin *et al.,* 2020; Kaland *et al.,* 2023; Long *et al.,* 2023); by linking these sources of information it may be possible to elucidate the sources of individual variability in scale cortisol and to partition stress-related signals. In addition, it is worth investigating if other hormones that are detectable in scales (Kennedy and Janz, 2022, 2023a) are preserved during storage. Ultimately, when relationships between scale concentrations and physiological processes are established, scale collections can be used to reconstruct individual responses to environmental change over extended time periods, to monitor changes in contemporary populations and to define physiological targets for conservation and management.

## Data Availability

Data are publicly available through the Zenodo data repository: https://zenodo.org/records/13235237.
